# Assessing Histological Inflammatory Activity in Patients With Ulcerative Colitis: A Diagnostic Accuracy Study Testing Fecal Biomarkers Lactoferrin and Calprotectin

**DOI:** 10.1093/crocol/otaa053

**Published:** 2020-06-27

**Authors:** Jost Langhorst, Lana Kairey, Angela Oberle, James Boone, Gustav Dobos, Hendrik Juette, Andrea Tannapfel, Andreas Rueffer

**Affiliations:** 1 Department of Internal and Integrative Medicine, Klinikum Bamberg, Bamberg, Germany; 2 Chair for Integrative Medicine, University of Duisburg, Essen, Germany; 3 Department of Internal and Integrative Medicine, Kliniken Essen-Mitte, Faculty of Medicine, University of Duisburg-Essen, Essen, Germany; 4 TechLab Inc, Blacksburg, VA, USA; 5 Institute for Pathology, Ruhr University Bochum, Bochum, Germany; 6 Enterosan, Labor L+S AG, Bad Bocklet-Großenbrach, Germany

**Keywords:** ulcerative colitis, inflammatory bowel disease, lactoferrin, calprotectin, sensitivity and specificity

## Abstract

**Background and Aims:**

Histological remission has arisen as the optimal treatment outcome in ulcerative colitis (UC). The aim of this retrospective study was to explore the diagnostic performance of the noninvasive fecal biomarkers calprotectin (FC) and lactoferrin (FL) compared to the histological indices Nancy Index (NI) and Riley Index (RI).

**Methods:**

This study is a retrospective diagnostic accuracy study based on secondary analysis of patient data from 2002 to 2017 extracted from medical registries of our clinics in Essen-Mitte, Germany. Patients with UC underwent a colonoscopy, with biopsies taken from the rectum and the sigmoid scored by 2 experienced pathologists according to NI and RI and provided a stool sample within 7 days pre- or post-colonoscopy. Diagnostic accuracy of recommended cutoffs for FC (>50 μg/g) and FL (≥7.25 μg/g) were tested against our *reference standard* (NI ≥2) in terms of specificity, sensitivity, positive predictive value, negative predictive value, and accuracy (effectiveness).

**Results:**

The number of patients with UC recruited was n = 226, aged 45.2 (SD 13.3). Histological indices were highly correlated (*r* = 0.980, *P* < 0.001). Fecal biomarkers correlated moderately with NI (FC: *r* = 0.383, *P* < 0.001; FL: *r* = 0.420, *P* < 0.001) and RI (FC: *r* = 0.395, *P* < 0.001; FL: *r* = 0.424, *P* < 0.001). Fecal biomarker concentrations were increased in patients with active histological disease (NI ≥2), median [IQR], FC 69.72 [20.07–254.38], FL 18.59 [6.06–44.42], compared to those with inactive disease (NI ≤1), FC 12.35 [3.89 – 32.16], FL 3.14 [0.75–11.05], *z* = −6.60, *P* < 0.001. Fecal biomarker concentrations differed significantly across NI grades 0–4 (FC: H_4_ = 45.2; FL: H_4_ = 47.5, both *P* < 0.001). Patients with grade 0 had significantly lower concentrations of fecal biomarkers than those with grade 3 (median; FC 10.94 vs 72.22; FL 2.30 vs 29.10; both *P* < 0.001) or grade 4 (FC 10.94 vs 67.00; FL 2.30 vs 27.64; both *P* < 0.001), as well as grade 2 for FC only (10.94 vs 56.22, *P* = 0.001). Concentrations were also lower in patients with grade 1 compared to those with grade 3 (FC 17.49 vs 72.22; FL 4.24 vs. 29.10; both *P* ≤ 0.001) or grade 4 (FC 17.49 vs 67.00; FL 4.24 vs 27.64; both *P* < 0.001).

Receiver operating characteristics area under the curve showed moderate diagnostic accuracy for both FC 0.76 (95% confidence interval [CI] 0.70–0.83) and FL 0.73 (95% CI 0.66–0.80). Optimized cutoffs for both FC (≥34.29) and FL (≥5.85 μg/g) had slightly improved accuracy, compared with the manufacturer’s cutoffs (FC: 69.9% vs 65.9%; FL: 71.7% vs 69.0%).

**Conclusions:**

Fecal biomarkers calprotectin and lactoferrin correlate with histological disease activity and differentiate between patients in histological remission from those with evidence of moderate to severe disease activity. Their noninvasiveness, in addition to being inexpensive, supports their use in the clinical monitoring of patients with UC.

## INTRODUCTION

Inflammatory bowel disease (IBD) refers to chronic diseases characterized by idiopathic, recurrent, and remitting inflammation of the gastrointestinal tract. The two diagnosable forms, ulcerative colitis (UC) and Crohn’s disease (CD), affect more than 0.3% of populations in westernized countries.^1^ In UC, inflammation is confined to the colon, leading to characterizing symptoms of abdominal pain, diarrhea, and rectal bleeding. Healing of the intestinal mucosa has been associated with long-term remission and minimizing the need for colectomy.^2^ Mucosal healing may be directly assessed visually via endoscopy and hence endoscopy remains the gold standard for routine monitoring of UC.^3^ More recently, however, histological remission has arisen as a superior treatment target in UC, given its greater performance in predicting future relapse or sustained remission in patients with UC, compared with endoscopy.^4–7^ Thus, it has been recommended that endoscopic assessment be complemented by a validated histological index for clinical monitoring of patients with UC.^8^

Histological scoring systems quantify the degree of inflammation, integrity of the epithelium, and alterations to the architecture of colonic crypts observed in tissue samples.^9^ While a number of scoring systems have been developed, only the Nancy Index (NI) has been validated for use in clinical practice and in clinical trials with patients with UC,^10^ although the Riley Index (RI) is more commonly applied in clinical practice.^11^ A significant limitation to the assessment of histological inflammation, however, is the need to collect tissue samples (biopsies) via colonoscopy. This invasive procedure is expensive and burdensome to patients.^12–14^ Valid, reliable, and less-invasive diagnostic methods are therefore crucial for routine assessment of disease status in patients with UC.

Fecal biomarkers provide a promising alternative for this purpose given their relative ease of collection and analysis, lower cost, and greater acceptance by patients, in comparison to colonoscopy.^15^ In our previous work, we showed 3 neutrophil-derived proteins (fecal calprotectin [FC], fecal lactoferrin [FL], and PMN elastase) were able to discern between active and remitting UC^16^ and that elevated levels are predictive of a future flare in patients with UC.^17^ FC, a calcium and zinc-binding protein found within the cytosol of innate immune cells (eg, neutrophils), correlates strongly with endoscopic disease activity^17, 18^ and has the utility of being able to detect early relapse (ie, prior to clinical symptoms) in patients with quiescent UC.^17, 19, 20^ Furthermore, 3 histological scoring systems the Geboes Score, NI, and Robarts Histopathology Index are predictive of FC levels in patients with UC.^21^ FL, an iron-binding glycoprotein found in polymorphonuclear neutrophils, has also been shown to be increased in active UC.^22–24^ As an inflammatory response is initiated prior to the onset of clinical relapse, this offers a window of opportunity to predict and prevent potential flares in patients with UC by detecting the presence of these biomarkers in the feces.^25^ Numerous studies support this, demonstrating an increase in both FC and FL in severe (or relapsing) UC.^26–29^ Furthermore, FC and FL both remain stable for up to 7 days when held at 4°C, further favoring their use for routine monitoring of disease activity in UC.^30, 31^

A limitation, however, to the use of fecal biomarkers in diagnosing histological disease activity in UC is the lack of defined cutoff values for grading the presence of histological disease activity or severity in practice. The aim of this retrospective diagnostic accuracy study was therefore to (1) examine associations between fecal biomarkers (*FC* and *FL*) and histological indices (*NI* and *RI*) and (2) evaluate the diagnostic performance of fecal biomarkers against *NI* as the reference standard for grading the degree of histological inflammation in UC.

## METHODS

The reporting of this study is in accordance with the 2015 *Standards for Reporting of Diagnostic Accuracy Studies* guidelines.^32, 33^ Ethical approval was granted by the ethical committee of the Faculty of Medicine, University Duisburg-Essen (No. 18-8268-BO). This study was conducted within the Clinic for Naturopathy and Integrative Medicine, Clinics of Essen-Mitte, Germany, in affiliation with the Faculty of Medicine, University Duisburg-Essen.

### Study Design

This was a retrospective diagnostic accuracy study based on the secondary analysis of clinical patient data extracted from medical registries between 2002 and 2017.

### Study Procedure

All inpatients and/or outpatients presenting to the Department of Internal and Integrative Medicine, Clinics of Essen-Mitte, Germany for UC treatment between 2002 and 2017 were invited to participate. Two research coordinators (A.O. and Dorit Schroeder) approached patients who were provided information about our department’s research. Interested patients gave their written consent to the use of their medical data for use in prospective research at the clinics in Essen-Mitte and were scheduled for a colonoscopy and stool sample collection. For this retrospective analysis, the physical and/or electronic medical records (iMedOne, Deutsche Telekom AG; Bonn, Germany) of consenting patients were reviewed by A.O. and Dorit Schroeder to identify patients meeting eligibility criteria for inclusion. Data extracted from eligible patients included age, gender, smoking behavior, UC diagnosis (ie, ulcerative proctitis, left-sided colitis, or extensive colitis), current medications, fecal biomarker concentrations (FC and FL), and data for both histological indices (NI and RI).

### Eligibility Criteria

We included patients aged 18–75 years, with a prior diagnosis of UC verified by endoscopy, histopathology, and the presence of clinical symptoms, eg, rectal bleeding or diarrhea, who had undergone a colonoscopy with 6 biopsies taken (3 each taken from the sigma and rectum) with a stool sample harvested and frozen at −20°C within the 7 days before, or after, colonoscopy to facilitate the procedure for the patients, and who had results for both fecal biomarkers (FC and FL) available. We excluded patients with preexisting or newly diagnosed mental and/or physical illness(es), alcohol or drug abuse, complete colectomy, tumor diagnosis in the past 5 years, as well as pregnant or breast-feeding women, and those following a special diet.

### Index Test: Fecal Biomarkers

As described in our previous papers,^16, 17^ and briefly reiterated here, fecal samples were collected in the morning of the appointment in the study center by patients using a disposable plastic bucket-type device which avoids contamination with the toilet water artifact and simplifies laboratory sampling. Samples were then frozen at −30°C and sent within 24 hours to our laboratory (Labor L+S AG, Bad Bocklet-Großenbrach, Germany) for immediate analysis. Fecal samples were tested for total concentrations of FC and FL using an enzyme-linked immunosorbent assay (ELISA) according to the respective manufacturers’ instructions (IDK Calprotectin ELISA from Immundiagnostik, Bensheim, Germany and LACTOFERRIN SCAN kit from TechLab, Blacksburg, VA). Results were calculated from a standard curve and reported in micrograms per gram. The following manufacturer’s recommended cutoffs were applied to define a positive result for FC (>50 μg/g) and FL (≥7.25 μg/g).

### Reference Standard: Histological Indices

Biopsies collected during colonoscopy were evaluated by two experienced pathologists who, blinded to the results of the fecal biomarkers, independently scored either the NI^10^ or the RI.^11^ In the case of divergent results, both pathologists discussed and established a final result. The RI grades the severity of histological disease activity based on 6 criteria: (1) acute inflammatory cell infiltrate, (2) crypt abscesses, (3) mucin depletion, (4) surface epithelial integrity, (5) chronic inflammatory cell infiltrate, and (6) crypt architectural irregularities.^11^ Each feature is graded as either none, mild, moderate, or severe to yield an overall score ranging from 0 to 18. The NI also grades the severity of histological disease activity, but on only 3 criteria: ulceration, acute inflammatory cells infiltrate (ie, the presence of neutrophils in either the lamina propria or epithelium), and chronic inflammatory infiltrate.^34^ Each criterion is sequentially assessed to yield an overall grade (grade 0: absence of significant histological disease activity; grade 1: chronic inflammatory infiltrate with no acute inflammatory infiltrate; grade 2: mildly active disease; grade 3: moderately active disease; and grade 4: severely active disease). Grades 2–4 are consistent with histologically active disease and predictive of future relapse in patients with UC.^35^

### Data Analyses

Data were exported to SPSS Statistics for Windows, version 26.0 (IBM Corp., Armonk, NY) for analysis. Statistical significance was set at 0.05. Descriptive statistics were computed to describe participating patients in terms of age, gender, smoking behavior, diagnosis (proctitis, left-sided colitis, or extensive colitis), and medication use. Fecal biomarkers were compared to histological indices using nonparametric Spearman’s rank correlations, as data for fecal biomarkers were highly skewed. We then compared fecal biomarkers concentrations between patients with evident histological disease activity (ie, NI ≥2) and those in histological remission (NI ≤1) using the nonparametric Wilcoxon–Mann–Whitney test, and across each grade of the NI (grades 0–4) using the nonparametric Kruskal–Wallis test. We explored the performance of our *index tests* (ie, fecal biomarkers FC and FL) in diagnosing our *reference standard* for histological disease activity (ie, NI ≥2), using the manufacturer’s recommended cutoffs (ie, FC >50 μg/g, FL ≥7.25 μg/g) by creating two-by-two tables and calculating measures of specificity, sensitivity, positive predictive value (PPV), negative predictive value (NPV), and accuracy (effectiveness). Corresponding confidence intervals (CIs) were calculated using the Clopper–Pearson method for specificity, sensitivity, and accuracy, and logit method for predictive values (PPV and NPV).^36^ Given the breadth of cutoffs used for FC in the literature, we also planned to test FC at concentrations more than 100 μg/g and more than 150 μg/g, although due to small cell sizes only results for more than 100 μg/g were reported. Receiver operating characteristics (ROC) curves were then computed for both FC and FL to define the area under the curve (AUC) with corresponding 95% CI, as well as optimized cutoffs based on Youden’s Index ([Sensitivity + Specificity] – 1), which yields the highest attainable sensitivity and sensitivity for a given cutoff. Diagnostic performance measures for these optimized cutoffs were also reported (specificity, sensitivity, PPV, NPV, and accuracy) for purposes of comparison to the manufacturer’s recommended cutoffs.

## RESULTS

### Participants

Complete data were available for n = 226 patients. Table 1 presents the key characteristics of participants who were aged on average 45.2 (SD 13.3) years and mostly female (60.2%), nonsmokers (90.3%).

**TABLE 1.** Characteristics of Participating Subjects

Characteristic	n	%
Age group		
18–30	38	16.8
31–44	59	26.1
45–59	95	42.0
60–74	34	15.0
Female gender	136	60.2
Smoking*		
Current smoker	21	9.7
Prior smoker	32	14.7
Never	164	75.6
Disease geography†		
Ulcerative proctitis	45	20.9
Left-sided colitis	91	42.3
Extensive colitis	79	36.7
Current medications‡		
Mesalamine	149	67.4
Steroids	51	23.1
Thiopurine	17	7.7
Biologics	15	6.8

*Excludes missing data for 9 patients.

†Excludes missing data for 11 patients.

‡Excludes missing data for 5 patients.

Compared with those patients without histological disease activity (NI ≤1), those with histological disease activity (NI ≥2) had similar disease geography (ulcerative proctitis 21.3% vs 20.7%, left-sided colitis 48.0% vs 39.3%, and extensive colitis 30.7% vs 40.0%, *χ*^2^ = 2.04, *P* = 0.360).

Table 2 presents the nonparametric intercorrelations between fecal biomarkers and histological indices. While rank correlations between histological indices were strong (*r*_s_ = 0.980), those between fecal biomarkers were more moderate (*r*_s_ = 0.473; Table 2). Fecal biomarkers also showed moderate correlations between histological indices, with FL being more strongly correlated with histological indices than FC (Table 2).

**TABLE 2.** Summary of Nonparametric Intercorrelations, Medians, and Interquartile Ranges of Fecal Biomarkers (Calprotectin and Lactoferrin) and Histological Indices (Nancy Index and Riley Index)

	Calprotectin	Lactoferrin	Nancy Index	Riley Index	Median	IQR
Calprotectin	—	0.473*	0.383*	0.395*	33.27	8.49–113.74
Lactoferrin	0.473*	—	0.420*	0.424*	10.35	2.15–36.58
Nancy Index	0.383*	0.420*	—	0.980*	11	3–16
Riley Index	0.395*	0.424*	0.980*	—	8	2–12

Another option for data illustration here.

*P < 0.001.

FC concentrations were significantly higher among those with evidence of histological disease activity (NI ≥2; median 69.72 [IQR 20.07–254.38]), compared to those without (NI ≤1; median 12.35 [IQR 3.89 – 32.16]); *z* = −6.60, *P* < 0.001. Comparatively, concentrations of FL were also significantly higher among those with histological disease activity (NI ≥2; median 18.59 [IQR 6.06–44.42]), compared to those without (NI ≤1; median 3.14 [IQR 0.75–11.05]); *z* = −5.70, *P* < 0.001).

**FIGURE 1.**
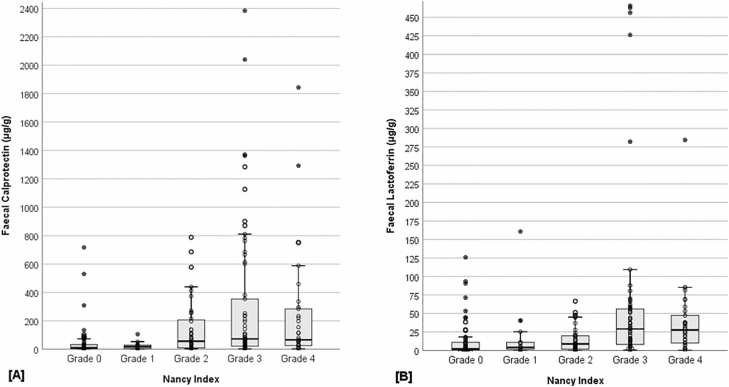
Boxplot illustration of fecal calprotectin (A) and fecal lactoferrin (B) concentrations across Nancy Index grades 0–4.

Fecal biomarker concentrations also differed across grades 0–4 of the NI (FC: H_4_ = 45.23, *P* < 0.001; FL: H_4_ = 47.51, *P* < 0.001) (Figure 1). Post hoc analyses revealed that patients with grade 0 histological disease activity had significantly lower FC concentrations, compared with grade 2 (*P* = 0.001), grade 3 (*P* < 0.001), and grade 4 (*P* < 0.001) (Table 3). Grade 1 concentrations were also significantly lower, compared with grade 3 (*P* = 0.001) and grade 4 (*P* = 0.011). Similarly, grade 0 had significantly lower FL concentrations, compared with grade 3 (*P* < 0.001) and grade 4 (*P* < 0.001). Grade 1 concentrations were also significantly lower, compared with grade 3 (*P* = 0.010) and grade 4 (*P* = 0.045). Those classified as grade 2 also had significantly lower FL concentrations compared with grade 3 (*P* = 0.003) and grade 4 (*P* = 0.038). Table 3 also illustrates the significant difference in the proportion of patients above the manufacturer’s recommended cutoffs for calprotectin (>50 μg/g) and lactoferrin (≥7.25 μg/g) across NI grades 0–4 (FC *χ*^4^ = 33.03, *P* <.001; FL *χ*^4^ = 32.74, *P* < 0.001).

**TABLE 3.** Distribution of Fecal Calprotectin and Fecal Lactoferrin Concentrations Across Nancy Index Grades 0–4

Nancy Index	N	Range	Q1	Q2	Q3	n (%)
Fecal Calprotectin						>50 μg/g
Grade 0	65	0.01–716.29	3.59	10.94	33.10	14 (21.5)
Grade 1	18	0.01–105.31	5.56	17.49	29.08	2 (11.1)
Grade 2	44	2.28–787.56	9.48	56.22*	206.15	22 (50.0)
Grade 3	65	1.28–2384.34	21.13	72.22* ^,^†	353.55	39 (60.0)
Grade 4	34	1.35–1843.31	25.32	67.00* ^,^†	283.95	21 (61.1)
Fecal Lactoferrin						≥7.25 μg/g
Grade 0	65	0.00–125.90	0.70	2.30	11.00	22 (33.8)
Grade 1	18	0.08–160.70	1.60	4.24	11.10	7 (38.9)
Grade 2	44	0.02–66.51	1.80	8.77	19.75	26 (59.1)
Grade 3	65	0.45–465.72	8.26	29.10* ^,^† ^,^‡	56.06	49 (75.4)
Grade 4	34	0.20–284.34	10.20	27.64* ^,^† ^,^‡	47.23	27 (79.4)

*Significantly different to grade 0 (*P* < 0.01).

†Significantly different to grade 1 (*P* < 0.05).

‡Significantly different to grade 2 (*P* < 0.05).

The diagnostic performance of FC and FL is illustrated in Table 4. The higher specificity and PPV for FC, compared with FL, arose from the relatively low number of false-positive diagnoses. In comparison, FL had a higher sensitivity, compared with FC, due to the relatively lower number of false-negative diagnoses (Table 4). A cutoff of more than 100 μg/g for FC showed improved specificity (92.8%) and PPV (90.6%), at the expense of a greater number of false negatives—reflected in the reduced sensitivity of 40.6%. Compared with the manufacturer’s recommended cutoffs, all parameters, except specificity, improved when using the optimized cutoffs for FC (≥34.29 μg/g) and FL (≥5.85 μg/g) (Table 5).

**TABLE 4.** Diagnostic Performance of Index Biomarkers (FC and FL) in Detecting Histological Disease Activity (ie, Nancy Index ≥2), With Corresponding 95% Confidence Intervals

		Nancy Index						
	Cutoff	Positive ≥2	Negative ≤1	Sensitivity %*	Specificity %†	PPV %‡	NPV %§	Accuracy %║
FC	Positive >50 μg/g	82	16	57.3 [48.8–65.6]	80.7 [70.6–88.6]	83.7 [76.4–89.1]	52.3 [46.9–57.7]	65.9 [59.4–72.1]
	Negative ≤50 μg/g	61	67					
	Positive >100 μg/g	58	6	40.6 [32.4–49.1]	92.8 [84.9–97.3]	90.6 [81.4–95.5]	47.5 [43.9–51.2]	59.7 [53.0–66.2]
	Negative ≤100 μg/g	85	77					
FL	Positive ≥7.25 μg/g	102	29	71.3 [63.2–78.6]	65.1 [53.8–75.2]	77.9 [72.0–82.8]	56.8 [49.3–64.1]	69.0 [62.6–75.0]
	Negative <7.25 μg/g	41	54					

*Sensitivity: proportion of patients correctly diagnosed as positive using the index test (TP/[TP + FN]).

†Specificity: proportion of patients correctly diagnosed as negative using the index test (TN/[TN + FP]).

‡Positive Predictive Value (PPV): proportion of patients correctly diagnosed as positive using the index test of all patients with a positive diagnosis using the index test (TP/[TP + FP]).

§Negative Predictive Value (NPV): proportion of patients correctly diagnosed as negative using the index test of all patients with a negative diagnosis using the index test (TN/[TN + FN]).

║Accuracy: proportion of patients correctly diagnosed as either positive or negative ([TP + TN]/[TP + FP + TN + FN]).

TP, true positive; TN, true negative; FP, false positive; FN, false negative.

**TABLE 5.** AUC Values for ROC of Index Biomarkers (FC and FL) With Optimized Cutoffs Based on Youden’s Index and Their Corresponding Sensitivity and Specificity

		Nancy Index						
	Optimized Cutoff	Positive ≥2	Negative ≤1	Sensitivity %*	Specificity %†	PPV %‡	NPV %§	Accuracy %║
FC	≥34.29 μg/g	93	18	65.0 [56.6–72.8]	78.3 [67.9–86.6]	83.8 [77.1–88.8]	56.5 [50.3–62.6]	69.9 [63.5–75.8]
	<34.29 μg/g	50	65					
FL	≥5.85 μg/g	109	30	76.2 [68.4–82.9]	63.9 [52.6–74.1]	78.4 [72.9–83.1]	60.9 [52.7–68.6]	71.7 [65.3–77.5]
	<5.85 μg/g	34	53					

*Sensitivity: proportion of patients correctly diagnosed as positive using the index test (TP/[TP + FN]).

†Specificity: proportion of patients correctly diagnosed as negative using the index test (TN/[TN + FP]).

‡Positive Predictive Value (PPV): proportion of patients correctly diagnosed as positive using the index test of all patients with a positive diagnosis using the index test (TP/[TP + FP]).

§Negative Predictive Value (NPV): proportion of patients correctly diagnosed as negative using the index test of all patients with a negative diagnosis using the index test (TN/[TN + FN]).

║Accuracy: proportion of patients correctly diagnosed as either positive or negative ([TP + TN]/[TP + FP + TN + FN]).

TP, true positive; TN, true negative; FP, false positive; FN, false negative.

**FIGURE 2.**
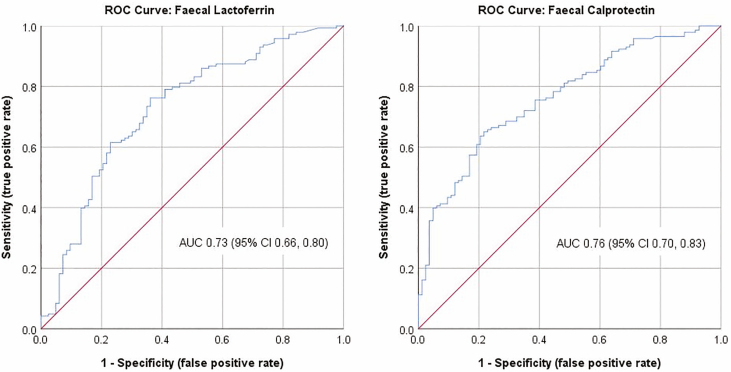
ROC curve for fecal calprotectin (FC) and for fecal lactoferrin (FL) with the AUC and associated 95% confidence interval.

Figure 2 illustrates the ROC curves for FC and FL, respectively, and AUC of 0.76 (95% CI 0.70–0.83) and 0.73 (95% CI 0.66–0.80), with 0.7–0.8 considered “good” in terms of diagnostic accuracy.^37^

## DISCUSSION

In this study, we first aimed to examine the relationship between fecal biomarkers FC and FL and histopathological indices NI and RI. While histological indices were perfectly correlated with one another, ranked correlations between fecal biomarkers and histological indices were moderate, albeit statistically significant.

Second, our study presents novel evidence for differentiation of FC and FL concentrations across grades 0–4 of the NI. We found that both FC and FL were able to differentiate between patients graded according to the NI with histological remission (grades 0–1) from those with evidence of moderate to severe histological disease activity (grades 3–4). FC and FL concentrations were notably higher in patients with grade 3 and grade 4 disease activity. However, concentrations were comparable between grade 3 and grade 4, suggesting that higher concentrations of both FC and FL are reflective of moderate to severe disease, rather than mild disease activity (ie, grade 2). The marked increase in both FC and FL in patients with grade 3 disease activity was unsurprising as grade 3 is designated where multiple clusters of neutrophils in the lamina propria and/or epithelium are present.^34^ As these fecal markers are released from neutrophils as part of our innate inflammatory response, these findings support the clinical appropriateness of using these biomarkers in the assessment of histological disease activity. Furthermore, FC and FL concentrations were slightly lower in patients with grade 4 disease activity—defined the presence of ulceration, which might be reflective of the initial clearing of these fecal markers from the intestinal lumen.

We further aimed to evaluate the diagnostic performance of fecal biomarkers against the NI which has been validated for use in the assessment of histological activity in UC in clinical practice and clinical trials. First, both FC and FL were both significantly increased in patients with evidence of acute inflammatory cells infiltrate (ie, NI ≥2)—a finding confirmed by Magro et al^21^ for FC levels among a sample of 377 patients with UC. Post hoc analyses showed a marked difference in FC and FL levels between NI grades 0–1 and 2–4, further supporting the appropriateness of these fecal biomarkers in assessing histological inflammatory activity in UC. These are supported by earlier research showing patients with UC with active histological inflammation, as measured by neutrophil infiltration, had significantly higher median levels of FC, compared to those without.^38, 39^ Our research is, however, the first to show this marked increase occurs also in levels of FL.

What is more, the diagnostic performance was achieved despite testing in a rather mild-to-moderate severity UC patient group with only 6.8% of patients were currently treated with biologics.

Using manufacturer-suggested cutoff values for calprotectin (>50 μg/g) and lactoferrin (≥7.25 μg/g), the proportion of patients with histological inflammation who tested positive to either calprotectin or lactoferrin (ie, sensitivity) was 57.3% and 71.3%, respectively. Alternatively, the proportion of patients in histological remission who tested negative to either calprotectin or lactoferrin (ie, specificity) was 80.7% and 65.1%, respectively. Arguably, it is the specificity of this test that is of greater value in clinical practice, as a high specificity reflects a higher proportion of patients without acute inflammatory cells infiltrate (ie, in histological remission) being diagnosed as such using the index biomarker. In this sense, FC with a specificity of 80.7% provides an inexpensive biomarker to allow clinicians to first “rule out” the presence of histological inflammation and avoid further invasive histological investigations via colonoscopy. Using the optimized cutoffs for FC and FL, specificity in both cases was lower than that obtained when using the manufacturers’ cutoffs, suggesting the manufacturer-recommended cutoffs of more than 50 μg/g for FC and at least 7.25 μg/g are superior for this purpose, at the expense of more false-negative diagnoses.

Overall, existing studies testing the diagnostic performance of fecal biomarkers against histological scoring indices are scarce. Both Theede et al^39^ and Guardiola et al^38^ tested the diagnostic performance of FC in predicting histological remission in patients with endoscopically quiescent UC, with reported AUCs ranging from 7.54 to 7.55, although sample sizes in these studies were comparatively small, reflected in the large CIs reported. Magro et al^40^ tested the diagnostic performance of FC and FL in predicting histological disease activity in patients with UC after receiving an 8-week course of infliximab. At week 8, the AUC (95% CI) for FC and FL was 0.94 (0.84–1.00) and 0.96 (0.88–1.00), both of which decreased after 1 year to 0.80 (0.58–1.00) and 0.81 (0.55–1.00).

Earlier studies compared tested the diagnostic accuracy of FC for defining endoscopically active disease using cutoffs ranging from ≥48 to ≥250 μg/g, with inconsistent sensitivities and specificities reported.^17, 19, 28, 29, 41–44^ Other studies have specifically examined the diagnostic accuracy of FC in predicting relapse, on the basis of clinical and endoscopic findings, in patients with UC, from which it has been concluded that the ideal cutoff for predicting relapse remains to be defined.^45^ Tested cutoffs above which FC is predictive of relapse in UC using in existing studies have ranged from 120 to 300 μg/g.^45, 46^

Comparatively, few studies have tested the diagnostic accuracy of FL for detecting endoscopically active disease. In our prior work, we showed cutoffs of more than 7.05 and at least 7.25 μg/g for FL to have a sensitivity of 92.6% and 75%, and a specificity of 66.7% and 62.5%, respectively, using the same IBD-SCAN method described in the present study.^17, 44^ Other studies adopted cutoffs for FL of at least 140 and more than 150 μg/g found to have a sensitivity of 67% and 62%, and a specificity of 68% and 65%, respectively, for predicting relapse in patients with UC.^29, 47^ Thus, suggesting a superior performance of FL as a marker of histological disease activity, as opposed to endoscopically active disease, when comparing these results to those reported in the present study.

Our results are not without their limitations. The number of biopsies collected from each patient was limited to 3 each from both the rectum and sigma which might lead to a sampling error. The primary strength of this study is the relatively large sample size compared to other studies in this field consisting of samples with less than 100 patients. Furthermore, as fecal biomarker concentrations are known to vary over the course of the day in calprotectin, we requested patients in this study to provide a morning stool sample only. FC and FL are also not the only fecal markers available that may be used in the assessment of intestinal inflammation, although they are arguably the most researched and used in clinical practice. For example S100A12 (calgranulin C) has shown greater specificity in predicting relapse in IBD compared to FC. In addition HMGB1 was correlated with disease activitity in pediatric and adult patients with IBS and Fecal Immunochemical Test has shown to be predictive of relapse in patients with UC.^49, 50^ Further the generalizability of our results is restricted to testing FC and FL concentrations using the specific laboratory testing kits described in the present study. Internationally, there are at least 6 different commercial testing kits available for measuring FC and FL concentrations, all offered from different manufacturers with some based on monoclonal ELISA and others on multiclonal ELISA.

As histological remission is predictive of relapse in patients with UC, even those in endoscopic remission, the assessment of histological disease activity in the clinical monitoring of UC is invaluable. Particularly as the presence of histological disease activity has been associated with the incidence of colorectal neoplasia and colectomy.^51–53^ Biomarkers of histological disease activity, such as fecal biomarkers, may be performed onsite permitting an inexpensive, noninvasive, and rapid measure of histological inflammatory activity in cases where information on the specific location and behavior of disease is not necessary. This being the primary limitation to fecal markers that once cannot concurrently screen for other lesions, as with colonoscopy, to support early detection of other intestinal conditions (eg, bowel cancer). A secondary limitation is that, as general markers of neutrophil infiltration into the intestinal mucosa, levels will also increase in response to intestinal infections and inflammation, such as undiagnosed celiac disease or infectious diarrhea.^16^

## CONCLUSIONS

Fecal biomarkers calprotectin and lactoferrin correlated with histological inflammation and differentiated between patients graded according to the NI with histological remission from those with evidence of moderate to severe histological disease activity. In addition, the fecal biomarkers are noninvasive and inexpensive which support their use in the clinical monitoring of patients with UC.

## Notes

Funding: This study was supported in part by a research grant from TechLab Inc, Blacksbury, VA, USA.

Conflict of interest (eg, employment, consultancies, stocks, honoraria, paid expert testimony, patent applications/registrations, and grants or other funding): J.L. has received research and travel grants from TechLab, Inc.; J.B. is an employee of TechLab, Inc.; other authors have no conflicts of interest to declare.

Data availability: The data that support the findings of this study are available from the corresponding author, J.L., on reasonable request.

Author contributions: J.L.: planning of the study, study design, data interpretation, and writing of the manuscript; L.K.: data interpretation and writing of the manuscript; A.O.: data interpretation; J.B.: study design and writing of the article; G.D.: data interpretation; H.J.: data interpretation; A.T.: data interpretation; A.R.: laboratory analyses, data interpretation, and final review of the manuscript; all authors reviewed and approved the final version of the manuscript.
